# Use of Microorganisms in the Recovery of Oil From Recalcitrant Oil Reservoirs: Current State of Knowledge, Technological Advances and Future Perspectives

**DOI:** 10.3389/fmicb.2019.02996

**Published:** 2020-01-17

**Authors:** Christina Nikolova, Tony Gutierrez

**Affiliations:** Institute of Mechanical, Process and Energy Engineering, School of Engineering and Physical Sciences, Heriot-Watt University, Edinburgh, United Kingdom

**Keywords:** microbial enhanced oil recovery, microorganisms, oil production, tertiary oil recovery, biosurfactants, enhanced oil recovery

## Abstract

The depletion of oil resources, increasing global energy demand, the current low, yet unpredictable, price of oil, and increasing maturity of major oil fields has driven the need for the development of oil recovery technologies that are less costly and, where possible, environmentally compatible. Using current technologies, between 20 and 40% of the original oil in a reservoir can be extracted by conventional production operations (e.g., vertical drilling), with secondary recovery methods yielding a further 15–25%. Hence, up to 55% of the original oil can remain unrecovered in a reservoir. Enhanced oil recovery (EOR) is a tertiary recovery process that involves application of different thermal, chemical, and microbial processes to recover an additional 7–15% of the original oil in place (OOIP) at an economically feasible production rate from poor-performing and depleted oil wells. EOR can significantly impact oil production, as increase in the recovery rate of oil by even a small margin could bring significant revenues without developing unconventional resources. Microbial enhanced oil recovery (MEOR) is an attractive, alternative oil recovery approach, which is claimed to potentially recover up to 50% of residual oil. The *in situ* production of biological surface-active compounds (e.g., biosurfactants) during the MEOR process does not require vast energy inputs and are not affected by global crude oil prices. Compared to other EOR methods, MEOR can be an economically and more environmentally friendly alternative. In this review, the current state of knowledge of MEOR, with insights from discussions with the industry and other stakeholders, is presented and in addition to the future outlook for this technology.

## Introduction

Global energy demand and consumption are expected to grow as fast-growing economies, such as China, India, and Brazil, are expected to account for over half of the increase in energy demand. In concert, there has been an increase in the demand for renewables (e.g., nuclear power, solar, wind, geothermal, wave, and biofuels) in recent years, although fossil fuels (oil, gas, and coal) will continue to remain as the dominant sources of energy and accounting for more than three-quarters of total energy supplies in 2035 (BP, 2017). Global proved oil reserves (over 1.6 trillion barrels of oil) have more than doubled over the past 35 years; however, 67% of the total petroleum reserves in the world comprise the residual crude oil in reservoirs that is difficult to recover (Shibulal et al., 2014). Using today’s technology, between 20 and 40% of the original oil in place (OOIP) in the reservoir can be recovered during a conventional production operation (e.g., vertical drilling). Secondary recovery of oil can achieve a further 15–25%, leaving behind up to 55% unrecoverable residual oil in oil reservoirs (Shibulal et al., 2014). The current low, yet unpredictable, price of oil, the increasing maturity of major oil fields (e.g., the North Sea), and the decreased number of newly developed oil fields are drivers to the industry for maximizing the efficiency of oil recovery in the current petroleum industry.

Enhanced oil recovery (EOR) is a tertiary recovery process that involves application of different thermal, chemical, and microbial processes to recover additional 7–15% of OOIP at an economically feasible production rate from poor performing and depleted oil wells (Bachmann et al., 2014). The impact of EOR on oil production can be significant given that an increase in the recovery of oil by a mere 1% translates to an impressive yield of 70 billion barrels of global oil reserves excluding the exploitation of unconventional resources (e.g., heavy crude oils, bitumen and tar sands) (Thomas, 2008). Microbial enhanced oil recovery (MEOR) is an alternative oil recovery approach which is claimed in the literature to be very promising in recovering up to 50% of residual oil (Shibulal et al., 2014; Geetha et al., 2018). MEOR processes involve the injection of indigenous or suitable exogenous microorganisms (mainly bacteria) together with nutrients into the oil reservoir to promote *in situ* microbial growth or by-production of microbial fermentation compounds that have the ability to influence the physico-chemical properties of crude oil and reservoir conditions to benefit oil production (Van Hamme et al., 2003; Voordouw, 2011). In this review, the current state of knowledge of using microorganism for EOR (i.e., MEOR), with insights from discussions with the industry and other stakeholders, is presented.

### Enhanced Oil Recovery (EOR)

**FIGURE 1:**
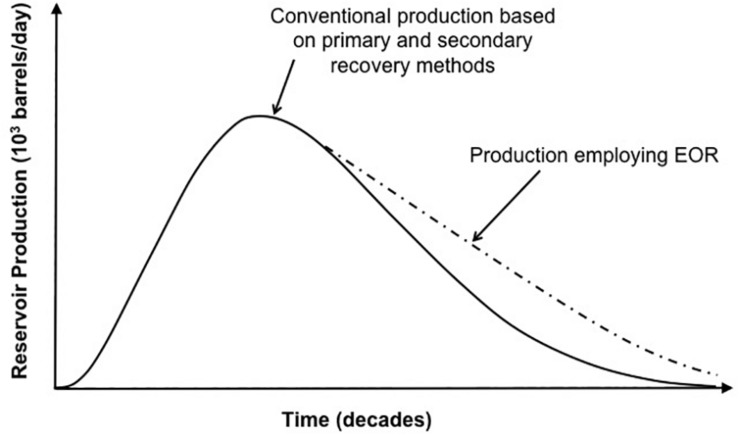
EOR effectiveness over time compared to primary and secondary production. Production curve in this graph represents general trend of production. A realistic production curve (not shown) may have many peaks and faults during the exponential production phase. Adapted from Nwidee et al. (2016).

Traditional crude oil production recovery (i.e., primary and secondary) efficiency is only 30–40% of the OOIP in the reservoir (Sen, 2010). Usually, the recovery efficiency decreases during the gradual depletion of light crude oil from a reservoir, leaving behind residual more viscous crude oil. “Tertiary” processes are required to improve the recovery of the residual oil. This tertiary process is referred to as EOR which can yield an additional recovery of 15–30% of oil in a reservoir. Generally, EOR involves injection of fluid into a reservoir producing an increase in the recovery of oil above that achieved solely from pressure maintenance when using gas and water injection, or the conventional secondary recovery method (Figure 1). The physico-chemical characteristics of the crude oil in the reservoir would determine which EOR method will be used. Traditionally, chemicals are added to the injected water in order to reduce the viscosity and/or the interfacial tension (IFT) of the oil. Other fluids (e.g., hydrogen or nitrogen gases, highly pressurized carbon dioxide) that have IFT values of <0.1 mN/m with the oil can also be directly injected into the reservoir (Muggeridge et al., 2013). The implementation of most EOR processes requires a large investment compared to a conventional water flood, and can be economically attractive for larger oil fields when the oil price is high.

### When Is EOR Used?

Oil and gas exist as fluids within the pore space of subsurface sedimentary rocks (typically sandstones or carbonates) and are produced by creating pressure gradients within the reservoir. After a well is drilled, the process when the first oil flows is called primary recovery which is facilitated by the reservoir pressure. It is the cheapest way to produce oil and accounts for 20% of the OOIP in reservoirs worldwide. Naturally, over time the natural pressure in the reservoirs decreases to a point where production is ineffective. In order to maintain the pressure and production rate, secondary recovery methods are used. Such methods typically include injection of water (*aka* water flooding) or gas (*aka* gas flooding) (Muggeridge et al., 2013). The injected fluids sustain the pressure, but they also sweep the oil trapped in smaller pores and encourage its flow toward the wellhead. Secondary recovery methods can be more expensive compared to the cost for operating the primary recovery process, but can recover an additional 40% of OOIP. Over time, secondary recovery becomes inefficient due to the high viscosity of the residual oil, low permeability of formation rock, high IFT between the aqueous phase and hydrocarbon, heterogeneities in the reservoir, and any number of other factors. Tertiary recovery, explicitly defined as EOR, offers new recovery methods that can address, to some extent, some of the problems that limit the full recovery of the oil in a reservoir (Bachmann et al., 2014).

### Types of EOR Methods

**FIGURE 2:**
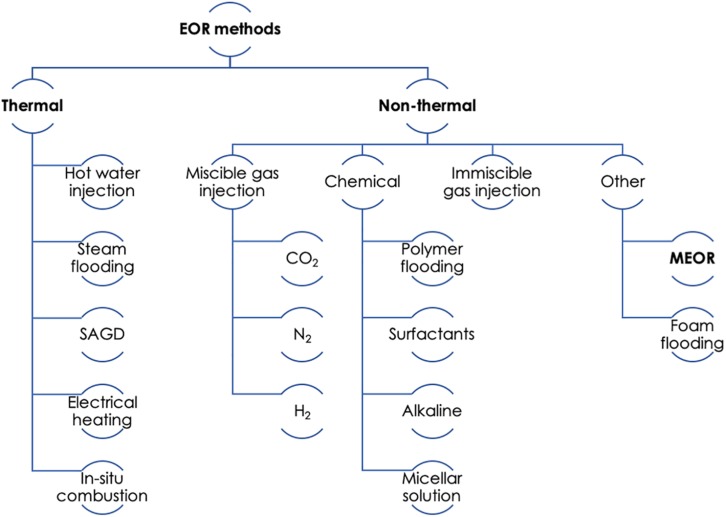
Summary of enhanced oil recovery (EOR) methods. SAGD, steam assisted gravity drainage; MEOR, microbial enhanced oil recovery. Adapted from Thomas (2008).

Enhanced oil recovery methods can be categorized under the broad classification of thermal and non-thermal, as illustrated in Figure 2. Thermal processes include hot water injection, steam flooding, and *in situ* combustion. In comparison, non-thermal processes are more diverse and include gas (CO_2_, N_2_, and stack gas) and chemical (polymers, surfactants, and alkali) injection, as well as MEOR (discussed below) and foam flooding. For lighter oils the oil recovery may be achieved by miscible gas injection, water alternating gas (WAG) injection, polymer or surfactant flooding, and changes in flow behavior. For heavy oils, thermal processes such as cyclic steam injection and *in situ* combustion would be more appropriate (Thomas, 2008). Thermal and gas injection (mainly CO_2_) methods are the most commonly used ones for sandstone reservoirs, mainly because CO_2_ is plentiful and cheap to obtain, and sandstones have been extensively tested at pilot and commercial scale (Alvarado and Manrique, 2010).

Thermal EOR projects have been concentrated mainly in regions with known heavy oil fields, such as in Canada, Venezuela, Brazil, China, and the United States. In particular, the Steam Assisted Gravity Drainage (SAGD) process is becoming an important method for increasing oil production in oil sands; however, commercial applications of SAGD are reported in Canada only. The *in situ* combustion method for recovery of heavy crude oils has also been considered as a viable option in past decades. However, this EOR process had not been fully accepted among oil field operators because there is a general lack of understanding of how the process behaves, which leads to inconclusive or failed pilot tests. Other thermal EOR methods, such as downhole steam generation, microwave technologies and electric or electromagnetic heating, have been proposed to have none or low impact on oil production but have not yet been proven to be technically and economically practical compared with traditional EOR thermal methods (Alvarado and Manrique, 2010).

Chemical EOR (cEOR) methods, and more specifically polymer flooding, were most popular in the 1980s, but since then their popularity steadily declined. Nevertheless, polymer and surfactant flooding are among the most commonly used cEOR methods (Demirbas et al., 2015). However, these methods are highly sensitive to volatility of oil price despite the development of more efficient and cheaper surfactants and polymers. There are some on-going large-scale projects involving polymer flooding in Argentina, Canada, China, India, and the United States (Alvarado and Manrique, 2010). Other cEOR methods, such as the injection of surfactant, alkali, alkali-polymer, alkali-surfactant-polymer, surfactant-polymer, and/or micelle solutions have been tested, but on only a limited number of fields. Hence, the lack of sufficient data on the successful application of these types of chemicals for cEOR limits their application due to their potential unpredictability.

Gas injection (miscible and immiscible) improves microscopic displacement efficiency by reducing the IFT between the oil and the displacing gas fluid. When used after a water flood, the injected gas helps in re-establishing a pathway for the remaining residual oil to flow through the rock space, which then leads to low residual oil saturation. The reservoir conditions, as well as local availability, would influence what type of gas is most likely to be injected in the reservoir. CO_2_, for instance, is miscible with oil at a relatively low pressure and temperature (shallow reservoirs) and is readily available. Injection of CO_2_ is most suitable for light to medium light oils (>30° API) in sandstone reservoirs (Thomas, 2008). Most CO_2_ flooding field projects are being conducted in the United States and Canada.

Thermal and chemical methods have some major disadvantages such as high energy requirement and chemical costs. In gas injection, high-pressure gas compressors are needed to convert the gases into liquid. The installation of gas compressors is expensive, and their availability needs to be considered. In addition, CO_2_ injections can cause corrosion of steel pipes, unless production facilities, including well casings, flowlines and pipelines, are designed to avoid this. Provision for the separation of CO_2_ from produced hydrocarbons also needs to be accounted for Gieg et al. (2011). Nitrogen requires a relatively high reservoir pressure and moderate temperatures (in deep reservoirs) for miscibility. Injection of nitrogen would also require the use of additional equipment to extract it from the atmosphere, therefore this method is rarely used. Hydrogen gas usually occurs naturally in the reservoir itself and, thus, most widely used. However, in most cases, the produced hydrogen gas, must be enriched artificially with heavier components to make it miscible or nearly miscible with the oil (Muggeridge et al., 2013).

## Microbial Enhanced Oil Recovery (MEOR)

### What Is MEOR?

**FIGURE 3:**
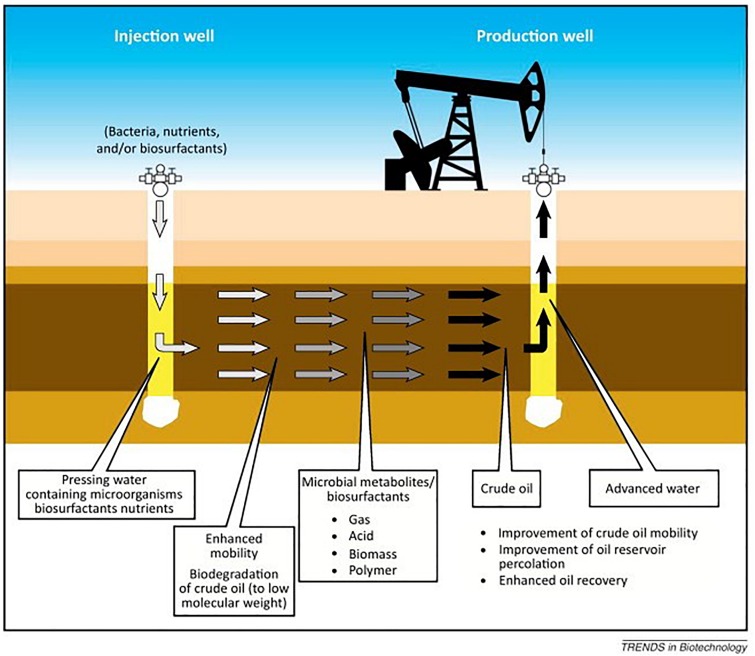
A schematic presentation of the MEOR process in an oil reservoir. Source: Marchant and Banat (2012). Reprinted with the permission by the authors.

An alternative approach for oil recovery is MEOR. MEOR often constitutes the introduction of live microorganisms with essential nutrients into an injection well. When favorable environmental conditions are present in the reservoir, the introduced microbes grow exponentially and their metabolic products mobilize the residual oil (Figure 3; Gao and Zekri, 2011). The injected microorganisms can produce a selection of metabolic products which find useful applications in EOR (Table 1). The growth of the microorganisms and their effects depend on any number of several factors (Bachmann et al., 2014), such as: (i) pressure, porosity and permeability, temperature, pH, dissolved solids, and salinity of the reservoir; (ii) availability of nutrients to the bacteria; (iii) the specific type of microorganisms injected into the reservoir. MEOR is believed to be able to extract up to 50% of the residual oil left in a reservoir after primary and secondary recovery processes have been exhausted (Sen, 2008). In general, this additional recovery is accomplished by modification of the chemical and physical properties of reservoir rocks and crude oil by the microbial growth and metabolites produced (Shibulal et al., 2014). MEOR could, therefore, get around the fundamental impediments to efficient oil recovery, such as high crude oil viscosity, low permeability of the reservoir, and high oil-water IFTs which produce capillary forces that are sufficiently high in retaining the oil within the pores of the reservoir rock.

**TABLE 1:** Microbial products and their application in MEOR.

**Product**	**Application in oil recovery**
Biomass	Selective biomass plugging, viscosity reduction, oil degradation, rock wettability alteration
Biosurfactants	Oil emulsification, decrease of interfacial tension, viscosity reduction
Biopolymers	Injectivity profile modification, mobility control
Solvents	Oil dissolution, viscosity reduction
Acids	Permeability increase, emulsification
Gases	Increased pressure, oil swelling, decrease of interfacial tension, viscosity reduction, permeability increase

Mixed microbial populations (principally bacteria and archaea) are commonly used as mixtures or formulations with metabolic products (e.g., solvents, acids or gases) to increase recovery and prolong the life span of the oil wells. Ideally, metabolic products are produced by the bacteria. For example, solvents such as acetone, butanol and propan-2-diol are produced by bacteria of the genera *Clostridium*, *Zymomonas*, and *Klebsiella*. Methane and hydrogen gas are produced by species of bacterial *Clostridium* and *Enterobacter*, as well as by the archaeon *Methanobacterium*. Fermentation gases can re-pressurize wells, leading to displacement of light crude oil in the well and, thus, facilitating its recovery (Van Hamme et al., 2003). Among distinct microorganisms used in MEOR, *Clostridium* are the most suitable because of their highly resistant endospores that enable survival at unfavorable conditions (Nwidee et al., 2016). Some *Bacillus* strains are also effective as they can lead to *in situ* production of biosurfactants which are favorable for the MEOR process (Joshi and Desai, 2013; Pereira et al., 2013). Nutrients, commonly in the form of fermentable carbohydrates, are injected to promote microbial metabolism.

### History of MEOR

In 1926, Beckman was the first to suggest that microorganisms could be used to mobilize oil from porous media, such as soil and rock. It wasn’t until the 1940s, when ZoBell and co-workers started a detailed systematic investigation to assess this. In his work, Zobell explained the leading processes accountable for oil release from porous media. These are: (1) dissolution of inorganic carbonates by bacterial metabolites; (2) generation of bacterial gases which decrease the viscosity of oil, and by that enhancing its flow through the rocks; (3) production of surface-active compounds by some bacteria; and (4) the high affinity of bacteria for solids, which would dislodge oil films from the surface of rocks in the reservoir. In 1947, Zobell defined and later patented different processes by which bacterial products (e.g., gases, acids, solvents, biosurfactants, and cell biomass), liberated oil from sandpack columns in laboratory tests (Lazar et al., 2007). In the 1960s and 1970s, significant research activity took place in former Czechoslovakia, Hungary, and Poland. The majority of field trials that had been conducted in these countries involved the direct injection of a pre-made consortium of facultative anaerobic bacteria (e.g., *Clostridium*, *Bacillus*, *Pseudomonas*, *Arthrobacterium*, *Micrococcus*, *Peptococcus*, *Mycobacterium*, and others) chosen for their ability to produce high quantities of bacterial products of interest. In 1958, Heinningen suggested the idea of selective plugging recovery, where oil recovery would be achieved from water floods by producing polysaccharide slime *in situ* from an injected microbial system based on molasses. The selective plugging recovery has been recognized as an important additional mechanism of oil release from reservoir rocks.

In the last three to four decades, the basic physiology and ecology of oil reservoir microorganisms has been demonstrated. Further to this, genetic engineering of indigenous microorganisms has advanced in recent years. It has since been established that cyclic microbial recovery, also known as single well stimulation, microbial flooding recovery, and selective plugging recovery are achievable applications, although conclusive data from large-scale field trials are scarce (Shibulal et al., 2014). In summary, the petroleum crisis in 1970s led to increased research efforts to scientifically evaluate MEOR as a recognized EOR method. Since then, many research projects were carried out all over the world in countries such as the Australia, Bulgaria, Canada, China, Germany, Norway, Poland, Romania, Russia, United Kingdom, and United States (Lazar et al., 2007; Town et al., 2010; Weidong et al., 2014; Li et al., 2015; Patel et al., 2015; You et al., 2016).

### Field Trials

During field trials, bacteria can be injected through both a production well and a water injection well through the annulus (Table 2). More than four hundred MEOR field tests have been conducted in the United States alone. However, the majority were single-well stimulation (also known as microbial “huff and puff”) treatments on low-productivity wells; hence, reliable data are sparse. Although single-well simulations can have benefits in some circumstances, some major operators, such as Shell, consider that in the MEOR process there has to be a field-wide impact (Shell Global Solutions, personal communication). On the other hand, smaller oil and gas companies will consider single-well stimulations if this is an important method by which they produce oil. MEOR may be attractive to independent oil producers, who mostly produce on average of less than half of a tonne of oil per day from “stripper wells,” the majority of which are found in the United States (Van Hamme et al., 2003). In Europe, MEOR field projects are mostly confined to Norway (Equinor) (Bødtker et al., 2009), Poland (RAM Biomedicals; Polish and Gas Institute) (Falkowicz et al., 2015) and Germany (BASF; Wintershall) (Alkan et al., 2014, 2016). In China, field trials only started in the last 10–20 years and show some promising, but mixed, results (Weidong et al., 2014; Li et al., 2015). Despite most published field studies reporting different MEOR treatments to being, to some extent, successful, full field-scale trials have as yet not been conducted. A general consensus about the economic feasibility of MEOR has, therefore, not been reached among oil operating companies.

**TABLE 2:** Comparison between microbial huff and puff operation, and bacteria flooding. Adapted from Gao and Zekri (2011).

**Microbial huff and puff**	**Bacteria flooding**
Bacteria injected through production tubing	Bacteria injected through injector well
Localized effect near the wellbore	Transport bacteria deep into the reservoir via water flooding
Reservoir shut-in period to allow bacteria to grow	Reservoir shut-in period to allow bacteria to grow
Repeat several times to maximize the gain	Large scale effect
Preferred MEOR option	Involve drilling of injector well unless some are present

### Advantages and Limitations of MEOR Technologies

As outlined below and summarized by Lazar et al. (2007), MEOR offers a number of advantages:

• The injected bacteria, nutrients and/or other natural products can be produced using inexpensive and easy-to-obtain raw substrates or even waste materials, and they are not affected by crude oil price compared with conventional cEOR processes.
• It is an economically attractive alternative for use in mature oil fields prior to their abandonment.
• No major alteration of the existing field facilities and infrastructure is required to apply the process, making it cheaper and easier to implement than another EOR method.
• Microbial processes consume less energy than thermal EOR processes.
• The process is primarily suited for carbonate oil reservoirs where some EOR technologies would not achieve desired results.
• The benefits of bacterial activity within the reservoir are amplified with time, whereas the opposite is true for other EOR technologies.
• Largely involves fully biodegradable products/additives and, hence, is considered a more environmentally compatible process.
• Microbial processes can be stimulated in situ within the reservoir, thus minimizing or eliminating the need to accommodate large storage facilities onsite/offshore.

It is worth noting that these advantages are based on the assumption that MEOR is successful in the field – i.e., the bacteria, nutrients, and/or bacterial products are uniformly distributed in the swept zone of the reservoir. Most of the published data on MEOR is based on laboratory tests where MEOR is shown to work. However, these results are rarely seen to replicate in the field due to general unpredictability of reservoir conditions, such as variable high temperature and pressure, multiphase flow of fluids in the reservoir, and uncertainty of the rock formation. The real potential of MEOR applications can only be fully assessed on field scale. As such, the real impact of MEOR has not yet been measured due to the apparent deficiency of both consistency in data collection and processing, and generation of quantitative data (regarding microbial processes *in situ*). Below is a summary of the various limitations to MEOR processes:

• It is a complex process because the desired bacterial activities depend on the physical and chemical characteristics of the reservoir.
• The majority of MEOR field projects are conducted on stripper wells, which renders MEOR a low incremental oil recovery process.
• It is a slower process than chemical or thermal EOR, and usually takes weeks or even months before any benefits are observed.
• MEOR is hard to control once implemented in the field, and its success is difficult to predict due to high heterogeneity of reservoirs.
• The cultivation of microorganisms in the laboratory that can grow and/or produce the desired metabolic products (e.g., biosurfactants) under reservoir conditions has proven difficult.

## Meor Mechanisms

There are two fundamental principles that MEOR is based on. In the first, oil movement through porous media is facilitated by altering the interfacial properties of the oil-water minerals – displacement efficiency (decrease in the IFT resulting to an increase in permeability), driving force (reservoir pressure), fluidity (miscible flooding; viscosity reduction), and sweep efficiency (selective plugging; mobility control). The second principle is known as upgrading and constitutes the degradation, but also the removal of sulfur and heavy metals from heavy oils, by microbial activity (Shibulal et al., 2014). However, the mechanisms behind oil mobilization are still poorly understood.

Gray et al. (2008) published a study in which they validated the different MEOR mechanisms that can achieve a reservoir-wide change in oil recovery (discussed further below). This study is by far the most comprehensive feasibility analysis of the most common MEOR mechanisms. These authors determined the microbial nutrient requirements for each mechanism, and performed a material balance to establish the oil recovery per nutrient mass for a typical sandstone North Sea petroleum reservoir with light crude oil. From a practical point of view, a mechanism must achieve the following goals: (1) a significant increase in oil recovery, and (2) an economically attractive yield ratio (incremental yield of oil divided by the input material for MEOR). If these requirements can be met, a mechanism(s) can be considered as promising for further assessment and research. It is important that each mechanism is feasible and robust in the field. This means that some basic reservoir data (i.e., net pay, porosity, permeability, pressure, temperature, stages of saturation and waterflood, viscosity, wettability etc.) is essential when considering which mechanisms could work best at the given reservoir conditions. If the yield ratio of a certain mechanism is near unity, the value of the oil would be comparable or less than the input materials, which would translate that mechanism(s) to having poor prospects for success in the field (Gray et al., 2008).

### Alteration of Interfacial Properties Between Oil, Water, and Rock

#### Reduction of Interfacial Tension (IFT) Using Biosurfactants

Biosurfactants produced *in situ* by microorganisms have been proposed as an effective mechanism to recover more oil from recalcitrant reservoirs (Banat, 1995; Brown, 2010). When poor oil recovery from oil wells is due to low permeability of the rock formation, or to the high viscosity of the crude oil, the ability of biosurfactants to reduce IFT between the flowing aqueous phase and the residual oil saturation can potentially improve the process efficiency and recover more oil (Banat et al., 2000). During the shut-in period, effecting an IFT reduction can significantly influence recovery of the oil during the initial stage, while later in the recovery process, both the produced biosurfactants and microorganisms – noting that microbial cells themselves can express IFT-reducing properties – can attach to the rock surface and alter its wettability (Daryasafar et al., 2016).

Biosurfactants can also potentially reduce the capillary forces that prevent oil from moving through rock pores. Viscous forces can promote flow in the reservoir, which is opposed by capillary force. A correlating parameter between the two forces, defined as the capillary number, is used to assess the likelihood of mobilizing the residual oil. More residual oil would be mobilized when the capillary number is large. A reduction in the residual oil will result in a higher recovery rate from the reservoir. Biosurfactants can increase the capillary number by reducing the IFT, but the decrease in IFT must occur by at least two orders of magnitude in order to mobilize the oil. Typically, IFT between hydrocarbons and water is between 30 to 40 mN/m. For biosurfactants to have any affect in MEOR, they must reduce IFT to 10^–3^ mN/m (Gray et al., 2008), which are values that have, to the best of our knowledge, not yet been reported. Furthermore, the process *in situ* is expected to result in the concentration of biosurfactants to be lower than required due to their dilution. As a result, the effectiveness of IFT reduction may be limited in practice.

The amount of biosurfactant required to recover oil depends on how much of the surfactant will absorb on the rock surface. Different rock formations have varying absorption values – sandstone, for example, is in the range of 0.1–1 mg surfactant per gram of rock, though this depends on the surface-active/IFT-reducing potential of the biosurfactant. For example, with the reservoir described by Gray et al. (2008), the authors calculated that if the residual oil saturation is 30% and the incremental oil recovery is 15%, only 23,000 m^3^ of incremental oil is recovered per 204,000 kg of surfactant. Hence, to achieve recoveries as high as 30–60%, flooding with biosurfactants would require very high injection volumes. In order to produce *in situ* biosurfactants, microorganisms need to utilize significant amounts of either the crude oil itself (primarily alkanes) or use exogenously added nutrients to power their activity, typically under the anaerobic conditions experienced in the reservoir. In cases where the indigenous microorganisms are left to “feed” on the alkanes, it is expected that the high mass of alkanes required to be metabolized for conversion into biosurfactants would significantly change the properties of the residual oil, such as increasing its viscosity by a factor of two or more, potentially making the recovery process difficult. From a material balance perspective, the microbial utilization of the crude oil to produce *in situ* biosurfactants seems very unattractive. Gassara et al. (2017) injected different concentrations of three carbon sources (molasses, acetate, and glucose) and nitrate into model upflow bioreactors to determine the effect of amount of carbon source on oil production. They established that to produce a significant amount of oil – up to 36% of residual oil in place – during incubation of nitrate-reducing bacteria *Pseudomonas* and *Thauera*, high concentrations of both the carbon source and nitrate were required – 17 mM of glucose/molasses or 57 mM acetate and 80 mM nitrate, respectively. Alternatively, if injection of nutrients (inclusive of a labile carbon source) is used to stimulate microorganisms to produce biosurfactants, it is expected that the nutrients would have to be injected regularly to flood the whole reservoir. In such case, however, other microorganisms not involved in biosurfactant production might consume the nutrients first, or compete with the biosurfactant-producing population, or they could consume the biosurfactant product itself.

#### Alteration of Wettability of Rock Formation by Microorganisms

Microorganisms and microbial by-products (e.g., biosurfactants) can, respectively, attach or adsorb to mineral surfaces, potentially altering the wettability of the rock. With respect to the microorganisms, in the oil reservoir they will grow solely at the oil-water interface – all life needs water, minimally, for growth. In sandstone formations, oil displacement is not fundamentally influenced by the wettability of the rock surface, and hence in this case wettability alterations by microbial activity is not a promising mechanism for enhancing oil recovery. In carbonate reservoirs, however, changes in the wettability (from oil-wet to water-wet) are much more significant for oil recovery because in a water-wet system, the oil tends to be in the large pores of the rock, while water is in small tracks and around the grains (Afrapoli et al., 2011). It may, thus, seem that water-wet conditions are favorable for increased oil recovery because oil is concentrated in larger pores and hence easier to access by flooding (Kowalewski et al., 2006).

### Changes in Flow Behavior

#### Microbial-Mediated Plugging of High-Permeability Zones

If a reservoir has high permeability zones or fractures between the injectors or producer, it is expected that the sweep efficiency would be poor. Usually, reduction of the effective diameter of the pore throat causes pore blocking, and also by large bacterial agglomerations, though with the latter this is less frequent. For reservoir rock formations with smaller pore throats, however, blocking by moving pieces of bacterial mass (cell agglomerations) is more important. Plugging of pores causes a diversion of the flow path (Kowalewski et al., 2006). The cell surface of oil-degrading bacteria is generally hydrophobic, thus conferring them with an affinity to adhere to oil droplets, as would occur in the reservoir. Bacterial growth occurs around the oil and, consequently, the bacteria will form a biofilm that can block large pores in the rock (Afrapoli et al., 2011). Blocking high permeability zones in the reservoir can also improve the areal sweep efficiency (improved conformance control) and lead to higher incremental recovery of the oil. In proportion to the volume of reservoir being treated, the highest ratio of oil that may be recovered is achieved by selective plugging of fractures. Smaller fractures can markedly favor the volume of incremental oil recovered compared to the volume of material injected (Gray et al., 2008).

#### Increase in Permeability by Organic Acid Production

The permeability of rocks indicates how easily fluids (oil and gas) will flow through the rock. High permeability (usually greater than 100 md; md is millidarcys, with darcy a measurement unit for permeability) will allow fluids to move rapidly through the formation. Reservoirs with lower permeability are expected to have decreasing production compared to reservoirs with high permeability, therefore improving the permeability and potential to lead to increased oil recovery. Production of organic acids (acetate, butyrate) by bacteria (e.g., *Clostridium* spp., *Enterobacter aerogenes*) *in situ* can dissolve formation rocks and open more pore volume, particularly carbonates, and as a result improve the permeability and fluids flow (Van Hamme et al., 2003). Work by Voordouw and co-workers at the University of Alberta patented a technique in which a microbial-stimulating fluid, consisting of hydrogen gas and/or carbon dioxide, can be used in carbonate heavy oil reservoirs. Indigenous microorganisms in the reservoir use the hydrogen as an electron donor and CO_2_ as an electron acceptor to produce acetic acid (via the Wood–Ljungdahl pathway), which in turn dissolves different carbonate compounds and consequently increases the porosity and permeability of the reservoir rock formation (Patent US20150053407; Table 3). However, a lot of acid would be required to dissolve significant amounts of rock in order to positively increase the pore volume. It is also possible that the bacterial cells may fill the pore volume themselves rather than improve the flow (Gray et al., 2008).

**TABLE 3:** Selection of patents related to MEOR technologies published since 2010. Source: espacenet (https://worldwide.espacenet.com).

**Patent name**	**Patent number (link)**	**Company/organization**	**Year**	**Country**	**Brief description**
Systems and methods of MEOR	US2012061117	Glori Oil Limited	2012	United States	Injection of microbes, treated water and oxygen supply into the oil-bearing formation
Microbes for viscosity reduction of heavy oil and process thereof	WO2017077553	The Energy and Resources Institute (TERI) and Oil and Natural Gas Corporation, Ltd.	2017	India	Injection of enriched population of microbial consortium comprising of a thermophilic microbe with accession number MTCC 5983 in heavy oil reservoir through water injection. Achieved 70–98% reduction of heavy oil viscosity and enhanced oil recovery by 15–30%.
Microbial enhanced oil recovery methods	MX343588	Geo Fossil Fuels LLC	2016	United States	Injection of genetically engineered halophilic microbe that contains functional genes for the metabolism of high-molecular weight hydrocarbons and lacks functional genes for the transport and oxidation of short chain alkanes at the cell membrane. Surfactant-producing genes are expressed even if a simple carbon source (e.g., molasses) is supplemented
Enhanced Microbial production of biosurfactants and other products, and uses thereof	WO2017044953	Locus Solutions LLC	2017	United States	Use of salt-tolerant over producing under anaerobic conditions *Bacillus subtilis* strains and their by-products, i.e., biosurfactants, to increase oil mobility and reduce paraffin build-up in the well-bore
Microbial enhanced oil recovery methods	MX2016002711	Geo Fossil Fuels LLC	2016	United States	Use of microbes selected or genetically modified to produce cell-free extracellular polysaccharide polymer without the formation of any significant bioplugging biofilm, which can reduce oil flow
Method for improving recovery ratio of indigenous microbial enhanced oil recovery	CN105201474	China Petroleum & Chemical Corp. and Petroleum Engineering Technology Research Institute China	2015	China	Method of improved oil recovery by endogenous microbial flooding with eutrophic activators (glucose, corn dry powder, and disodium hydrogen phosphate) in order to maximize the concentration of microorganisms in the reservoir
Composition and method for inhibition of SRB^∗^ in MEOR.	EP3178903	Wintershall Holding GmBH	2017	Germany	Injection of a specific blend of nutrients into the oil reservoir to stimulate indigenous microbes to decrease oil viscosity and inhibit SRB activity
Mixed bacteria producing biosurfactant and screening method of mixed bacteria	CN104877928	Suzhou ZFA New Energy Technology Co., Ltd.	2015	China	Identified mixed bacterial consortium consisting of *Trichoderma reesei*, *Pseudomonas stutzeri*, and *Lactobacillus rhamnosus* producing biosurfactant for enhanced oil recovery applications
Enhanced oil recovery and environmental remediation	WO2016122332	Statoil Petroleum	2016	Norway	Injection of one or more of nine novel bacterial strains including *Geobacillus toebii*, *Aeribacillus pallidus*, and *Anoxybacillus beppuenis* to an oil reservoir. The bacterial strains have been shown to grow on and produce compositions having biosurfactant-like properties from a crude oil substrate under conditions of pH, pressure, temperature, osmolality and oxygen concentration representative of an *in situ* subterranean oil reservoir
A steady state anaerobic denitrifying consortium for application in *in situ* bioremediation of hydrocarbon-contaminated sites and enhanced oil recovery	GB2511673	Du Pont	2014	United States	Inoculation a consortium of microbial species comprising at least one *Thauera* strain and at least two other strains from the group consisting of *Bacteroides*, *Azoarcus*, *Pseudomonas*, *Azotobacter*, *Clostridium*, *Anaerovorax*, *Finegoldia*, *Spirochetes*, *Deferribacter*, *Flexistipes*, *Chloroflexi*, and *Ochrobactum* species into anaerobic reservoir injection water, and injecting the resulting mix into an oil reservoir together with dissolved nitrate
Method of producing biosurfactants	WO2013110132	GFS Corp. AUS PTY Ltd.	2013	Australia	Use of vinasse, a by-product of the sugar industry, as a carbon substrate for the production of biosurfactants from *Bacillus* sp., bacteria
*In situ* microbial oxygen generation and hydrocarbon conversion in a hydrocarbon containing formation	WO2011076925	Shell International Research	2011	Netherlands	Injection of a water comprising an oxidizing compound (e.g., H_2_O_2_, NaClO_4_, KClO_4_, and NaNO_3_) into the oil reservoir to assist microbial chlorate reduction and thus produce *in situ* oxygen and stimulate indigenous thermophilic microbial activity using hydrocarbons as carbon and energy source. Microbial chlorate reduction requires temperatures no lower than 120°C
Microbial enhanced treatment of carbonate reservoirs for *in situ* hydrocarbon recovery	US20150053407	University of Alberta	2015	Canada	Injection of microbial stimulation fluid that includes H_2_ and/or CO_2_ and indigenous microbial cultures (including *Clostridium* and *Acetobacterium*) that converts at least a portion of the H_2_ and CO_2_ into a bioacid such as acetic acid. The bioacid promotes the dissolution of carbonate compounds in the reservoir which in turn increases the porosity and permeability of the carbonate reservoir to increase heavy hydrocarbon recovery
Methods for microbially enhanced recovery of hydrocarbons	US20180135393	UTI LP	2018	Canada	Injection of low molecular weight hydrocarbon (i.e., toluene) and nitrate to boosts indigenous microbial cultures ability to oxidize toluene and reduce nitrate in order to increase production of heavy oil

*^∗^SRB, Sulfur-reducing bacteria.*

#### *In situ* Production of Microbial Gas and Solvent

ZoBell first proposed the idea of using microbial gas production for MEOR, with the key concept of injecting or encouraging indigenous bacteria to produce CO_2_ and/or methane to restore the pressure in the reservoir to some extent, decrease oil viscosity, and dissolve calcite and siderite in limestone or carbonaceous sandstone to mobilize adsorbed oil (Zobell, 1946). CO_2_ flooding leads to the dissolution of sandstone components under field conditions. In under-saturated reservoirs, some microbial gas is likely to dissolve in the oil and reduce its viscosity. In addition, gas dissolved in the residual oil causes the oil to swell and consequently flow easier. However, it seems rather unlikely that bacteria would produce enough CO_2_ in naturally anaerobic conditions to achieve this (Bachmann et al., 2014). Low-pressure zones in the reservoir will allow compressed CO_2_ to expand, which will cause a drop in the temperature of the reservoir. Depending on initial temperature and pressure, this could potentially induce the precipitation of the paraffin/asphaltene fraction with potential adverse effects on oil recovery (Bachmann et al., 2014).

Microbial solvent production *in situ* has also been considered as a promising MEOR mechanism. Solvents are usually used to reduce the viscosity of the crude oil. A microbially generated liquid solvent, such as butanol, ethanol or acetone, produced by bacteria such as *Clostridium acetobutylicum*, *Clostridium pasteurianum*, or *Zymomonas mobilis*, would separate between the oil and water phases in the reservoir (Van Hamme et al., 2003). The fraction that dissolves in the oil may increase its mobility by reducing the oil viscosity. This would be economically attractive, but only if the difference in viscosity between the oil and the solvent is large enough to stimulate significant reduction in oil viscosity. Otherwise, it would take vast volumes of solvent to be injected in the reservoir in order to achieve even the slightest increase in oil recovery (Gray et al., 2008).

## Application of Biosurfactants for Meor

There are three methods through which biosurfactants promote oil displacement and movement in oil-bearing rocks: (1) reduction of IFT between oil-rocks and oil-brine, (2) changing the wettability of rocks, and (3) emulsification of crude oil (Sen, 2010). Methods 1 and 2 are covered in section “Alteration of Interfacial Properties Between Oil, Water, and Rock” above. Furthermore, the production of biosurfactants can contribute to the microbial metabolism of viscous oils, in turn resulting in the formation of lighter hydrocarbons which makes the oil more fluid. A number of specific characteristics of biosurfactants can influence MEOR (Gao and Zekri, 2011), as follows:

• Shear stability
• High solution viscosity
• Compatibility with reservoir brine
• Stable viscosity over a wide range of pH, temperature and pressure
• Sufficient reduction of the IFT between the oil and water.
• Low adsorption to formation rock; here, the tendency of surfactant to rock surface results in large mass requirements for mobilization of the residual crude oil.

### Biosurfactant Application Strategies

#### Injection of Biosurfactants Into the Reservoir

The option of injecting biosurfactants into a reservoir is not yet economically sustainable because the costs for industrial-scale production of biosurfactants are quite high and thus uneconomical. Such costs include bioreactor maintenance and the extraction of the biosurfactant(s) and their purification. This is often compounded by the fact that the production of microbial biosurfactants results in uneconomically low yields (e.g., 1–10 g/l from by bacteria). Yet, experimental evidence supports the efficacy of the biosurfactant flooding technique, and large multi-national research projects, such as MARISURF^1^ are exploring ways to optimize and increase yields of powerful surfactants/emulsifiers produced by novel species of marine bacteria. Biosurfactants have been shown to be as effective as conventional chemical surfactants or even better. Lichenysin, for example, is a very powerful biosurfactant synthesized by *Bacillus licheniformis* strain JF-2, isolated from well injection water and recently reclassified as *Bacillus mojavensis* JF-2 (Folmsbee et al., 2006). Lichenysin can reduce IFT to values of less than 10^–2^ mN/m (even at low concentrations (10–60 mg/l). In addition, this biosurfactant has been shown to be stable in temperatures as high as 140°C, a pH range from 6 to 10, salinity up to 10% w/v NaCl, and calcium (as CaCl_2_) concentrations of up to 340 mg/l (McInerney et al., 1990). In core flooding experiments, partially purified lichenysin recovered up to 40% of residual oil from sandstone cores compared to 10% recovery when chemical surfactants were applied (McInerney et al., 1990). Another *Bacillus* species, *Bacillus subtilis* produces another powerful biosurfactant called surfactin (Chandankere et al., 2014). Surfactin can reduce the ST to 26 mN/m of oil-water interfaces at concentrations as low as 0.005% (Mulligan, 2005; Al-Wahaibi et al., 2014) and enhance oil recovery from packed sand columns by up to 90% (Makkar and Cameotra, 1997).

Addition of biosurfactants during chemical surfactant flooding can also improve the flooding performance in general. It has been shown that in the presence of rhamnolipids, which are low-molecular-weight surfactants commonly produced by *Pseudomonas aeruginosa*, the adsorption to sandstone of the surfactant alkylbenzene sulfonate was reduced by 25–30%. Consequently, the quantity of oil recovered increased by 7% with the use of biosurfactants to the flooding solution. It has been suggested that rhamnolipids act as sacrificial agents by preferably adsorbing to the oil sands, making the surfactant more available for displacement activity and resulting in altering the wettability of the porous media (Perfumo et al., 2010). Macromolecular biopolymers, or high-molecular-weight bioemulsifiers, can be more effective than the low-molecular-weight biosurfactants (Sałek and Gutierrez, 2016). A typical example is emulsan produced by *Acinetobacter venetianus* RAG-1. Emulsan was shown to remove up to 98% of pre-adsorbed crude oil to limestone core samples, even at low concentration of 0.5 mg/ml (Gutnik et al., 1983). Recently, another biopolymer produced by *Rhizobium viscosum* CECT 908 was evaluated for its suitable application for MEOR (Couto et al., 2019). The biopolymer showed better efficiency than xanthan gum (a well-known biopolymer) in the recovery of heavy oils achieving just above 25% of additional recovery.

#### Injection of Laboratory-Cultivated Biosurfactant-Producing Microorganisms

The possibility of injecting biosurfactant-producing bacteria along with nutrients into a well to allow their growth and activity has been the most tested MEOR method. However, bacteria must grow and be metabolically active in regular reservoir conditions, which are regarded as extreme in terms of high temperature and pressure. As such, indigenous strains from reservoirs would be the ideal candidates over extremophilic microorganisms that have been isolated from other environments (Miyazaki et al., 2012). Indigenous reservoir bacteria can be incubated under reservoir conditions in large batches in the laboratory and subsequently be injected into the reservoir. For example, *B. mojavensis* JF-2 can grow while producing lichenysin under both aerobic and anaerobic conditions and at relatively high temperatures (e.g., 40°C) that are typical in oil reservoirs. Injection into oil-bearing rock formations and being as part of microbial consortium are the two possible applications considered for exploiting strain JF-2. It has been shown that flooding with *B. mojavensis* JF-2 achieved 14% rise in oil production, but also that living cells of the strain are able to survive up to 6 weeks after injection (Bryant, 1990). *B. licheniformis* was isolated from Zilaei oil reservoir in southwest Iran and shown to grow on crude oil at 50°C and produce a glycolipid biosurfactant. This organism was injected in sandstone core samples and allowed to grow and excrete biosurfactants for 7 days. The amount of oil recovered in this stage was almost 14% of OOIP (Daryasafar et al., 2016). Though these studies are limited, the potential of this direct injection approach is promising, but requires more testing. The amenability of indigenous reservoir bacteria to be cultivated in the lab is also a limiting factor, as few have ever been cultivated.

Using *in situ* MEOR, bacteria inoculated into the water that floods the well will initially progress into high permeability zones. Later, as the bacterial population grows, they can potentially clog those zones due to the increased number of bacterial cells, their size and cell surface negative charge. Consequently, this process leads to increased sweep efficiency, and higher oil recovery can be achieved. MEOR can replace cEOR, which is comparatively a more costly technology compared to MEOR, by using analogous compounds synthesized directly by microbes. A further advantage of microbially produced compounds for MEOR is that they are biodegradable and commonly associated with low toxicity (Banat et al., 2010).

#### Use of Nutrients to Stimulate Biosurfactant Production *in situ*

For an effective stimulation of biosurfactant production *in situ*, nutrients could be injected directly into oil reservoirs. However, this method is based on the assumption that oil reservoirs harbor their own indigenous population of microorganisms that are able to grow or survive under the extreme conditions (i.e., anoxic, high pressure, high salinity, high temperature) of the reservoir (Banat, 1995). The existence of such indigenous microbial ecosystems is still not well described or even fully understood because representative samples are hard to obtain and *in situ* analyses cannot be done easily. Even when samples are collected from a reservoir for analysis and experimentation *ex situ*, the behavior and activities of the microorganisms in a laboratory setting may not be a perfect representation to that in the reservoir. Technologies for injection of nutrients, in the form of carbon substrates and minerals, to stimulate the indigenous microbial communities in the well have long been known and are commercially available (Sunde, 1992; Falkowicz et al., 2015; Patel et al., 2015; Chen et al., 2017; Suri et al., 2019). Tracking the microbial response over time after an injection of nutrients can be conducted using 16S rRNA gene sequencing, metagenomics or metatranscriptomics. However, the scientific observation of *in situ* activities is difficult and it is nearly impossible to have untreated controls available for comparison due to exogenous contamination during the initial drilling process when nutrients and/or microorganisms can be introduced unintentionally in the reservoir by the drilling fluids. Youssef et al. (2007) confirmed that exogenous contamination of the reservoir was the likely reason for the presence of biosurfactant-producing bacteria in a nutrient-stimulated oil well. As a result, in wells treated with only nutrients, it was reported that no significant IFT reduction activities were detected.

## Biotransformation of the Oil *in situ* to Enhance Recovery

Of the various classes of crude oils, heavy crude oil is relatively viscous compared to other oils and does not flow easily due to its high density and specific gravity. The composition of heavy oils constitute a large percentage fraction of asphaltenes and resins, and is referred to “heavy” because of the high ratio of aromatics and naphthalenes to paraffins (linear alkanes) and high amounts of nitrogen, sulfur, oxygen, and heavy metals (Shibulal et al., 2014). Its formation as a heavy oil is due to lighter oils being exposed to bacteria, which degrade the lower-molecular-weight hydrocarbons in the oil, resulting in an oil with a higher content of more complex and higher-molecular-weight counterparts (Head et al., 2010). To decrease the viscosity of heavy oils in order to improve their recovery from a reservoir, at least two microbial mechanisms can be used: (1) microbial conversion of heavy to light oil components, and (2) microbial production of bio-molecules (e.g., biosurfactants) that alter the physical properties of the oil, such as reducing its IFT. For example, under anaerobic conditions *B. subtilis* was shown to degrade long-chain *n*-alkanes (>C_27_), with the percentage of *n*-alkanes of chain length <C_25_ becoming increased relative to untreated controls. Since most oil reservoirs are anoxic or anaerobic, under such conditions the microbial degradation of long-chain *n*-alkanes would be more relevant and realistic for MEOR.

Anaerobic hydrocarbon degradation, however, can cause serious problems in the reservoir. During secondary extraction of oil from the reservoir by injection of sea water, sulfur-reducing bacteria (SRB) present in seawater may enter the reservoir and cause “souring” of the petroleum by reducing sulfate to sulfide (Heider et al., 1999). Sulfide is highly corrosive to pipelines and storage tanks, even in the absence of oxygen, and poses a health hazard to humans. Significant evidence exists supporting that crude oil biotransformation happens *in situ*, however, there is not enough quantitative data to support improved oil recovery. Bacteria can also biotransform precipitated paraffin and asphaltene fractions from the crude oil. These waxy high-molecular-weight fractions of crude oil cause blockage of main drainage routes and pipelines, so their bacterial-mediated degradation is advantageous to the oil industry.

The aerobic biodegradation of hydrocarbons by bacteria starts with the oxidation of the oil via the activity of oxygenases. Alkanes are catalyzed into carboxylic acids and further broken down by β-oxidation, which is the principle metabolic pathway of lipids; the resultant formation of acetate subsequently enters into the tricarboxylic acid (TCA) cycle of central metabolism (Rojo, 2009). Conversely, the biodegradation of mono- and polycyclic aromatic hydrocarbons proceeds via hydroxylation of the ring moiety by the activity of dioxygenases; this leads to the formation of ring diols (catechols) that subsequently become cleaved and further degraded to acetate that enters the TCA cycle of central metabolism. The complete biodegradation (i.e., mineralization) of hydrocarbons results in the formation of carbon dioxide and water as end products, as well as cell biomass (Shibulal et al., 2014).

## Promising Microbial Species and Their Characteristics for Meor

Microorganisms selected for *in situ* MEOR applications must meet the most important requirement, which is, ideally, they should be able to degrade hydrocarbons and be able to survive and produce the desired metabolic products in the reservoir. Ideal candidates that meet these requirements are the indigenous microorganisms (primarily bacteria) in the reservoir. Microorganisms with the ability to degrade hydrocarbons are commonly referred to as hydrocarbonoclastic. The indigenous community of hydrocarbonoclastic bacteria in a reservoir would be expected to have a selective advantage over a microorganism or microbial consortium that is of exogenous origin because the former will be better adapted to the conditions in the reservoir. The main adaptations that microorganisms must have are high tolerance to high temperatures, salinity and pressure, as well as be active under anaerobic conditions (Ibrahim and Al-Maghrabi, 1999). Simulating high pressures in laboratory conditions is extremely difficult and few studies are found in the literature that have measured the tolerance of microorganisms to extreme pressures.

Indigenous microbial community structures in oil reservoirs is expected to vary as each reservoir is different in terms of depth, temperature, pressure, salinity and other characteristic features. Most studies exploring microbial communities use culture-based methods to recover and identify individual oil-degrading isolates and do not provide complete information of how these communities are structured. Nevertheless, as a general “rule of thumb” thermophilic anaerobic bacteria (*Thermotoga*, *Thermoanaerobacter*, *Thermodesulfobacterium*), hyperthermophilic archaea (*Thermococcus*, *Thermofilum*) and hydrogenotrophic methanogenic archaea (*Methanothermococcus*, *Methanobacterium*, *Methanoculleus*) are commonly the predominant microorganisms found in high temperature oil reservoirs and their diversity might be reduced at higher temperature (>80–90°C) and salinity conditions (>100 g/l) (Orphan et al., 2000, 2003; Dahle et al., 2008; Lin et al., 2014). Mesophilic bacteria, such as *Clostridium*, *Pseudomonas*, *Arcobacter*, *Geobacter*, *Desulfuromonas*, *Marinobacter*, and others, can also be found in high abundances in lower temperature oil reservoirs (Hubert et al., 2012; Zhang et al., 2012; Okpala et al., 2017). However, these organisms are likely introduced into the reservoir during drilling and water-flooding.

The main metabolic pathways associated with oil reservoir microbial communities are methanogenic, sulfate- and nitrate-reducing pathways under anaerobic reservoir conditions. The methanogenic pathway involves the conversion of a restricted number of substrates, such as hydrogen, CO_2_ and acetate to methane (Jones et al., 2008). Microbes utilize methanogenic metabolic pathways to biodegrade oil, and over geological time scales this results in the formation of heavy oil deposits. Sulfate-reducing microorganisms utilize sulfate and sulfite, as well as hydrocarbons, as terminal electron acceptors to reduce these inorganic molecules to hydrogen sulfide gas which can cause souring of crude oil under thermophilic conditions (Barton and Fauque, 2009). A common control measure for oil souring is to inject nitrate which is then reduced to nitrite by nitrate-reducing bacteria in the reservoir (in zones where temperature ranges between 45 and 65°C) and sometimes to dinitrogen (at reservoir temperatures up to 45°C) (Okpala et al., 2017). Nitrate is a powerful metabolic inhibitor of sulfate-reducing microorganisms. Continuous injection of sulfate-containing, nitrate-amended water can create clearing zones in the reservoir where nitrate-reducing bacteria are limited to the wellbore, and SRB to deeper areas in the reservoir (Okpala and Voordouw, 2018).

### Thermo-Tolerant Microorganisms

In most developed petroleum reservoir conditions, temperatures are expected to vary greatly but can be as high as 70°C, and even-100°C is some cases. In order to survive such high temperatures, thermophilic organisms are often spore-forming and possess thermally stable enzymes that allow normal functioning of cellular processes under such extreme conditions. Examples of such enzymes and their characteristics and functionalities are extensively described in the literature (e.g., Demirjian et al., 2001; Vieille and Zeikus, 2001; Gomes et al., 2016). *P. aeruginosa* incubated at 37°C produces rhamnolipids, which at a concentration of 1 mg/ml this biosurfactant has been shown to recover 22% of oil from sand-packed columns at 40°C, and to be more effective than the chemical surfactant Petrostep (15.6% oil recovery) (Gudiña et al., 2015). *B. subtilis* and *B. licheniformis* strains have been repeatedly isolated from many oil reservoirs, as well as from oil-contaminated sites (Cooper et al., 1981; Al-Wahaibi et al., 2014; Al-Sayegh et al., 2015; Daryasafar et al., 2016). Thermophilic hydrocarbon degraders of the genera *Bacillus*, *Thermus*, *Thermoanaerobacter*, *Thermococcus*, and *Thermotoga* have been isolated from high temperature reservoirs in China (90°C; Lin et al., 2014), California (80–90°C; Orphan et al., 2000) and North Sea (70°C; Dahle et al., 2008), and would therefore be ideal candidates for MEOR (Kaster et al., 2009). Three thermophilic hydrocarbonoclastic species of *Bacillus*, *Geobacillus*, and *Petrobacter* were reported to tolerate 55°C under strictly anaerobic conditions and to degrade hydrocarbons (Shibulal et al., 2014), suggesting their suitability for MEOR.

### Halo-Tolerant Microorganisms

Many species of bacteria have been shown to be halophilic (able to grow and thrive in relatively high salt concentrations), some of which with the added ability to also grow under the conditions experienced in oil reservoirs (Head et al., 2014). Strains of *B. subtilis* and *B. licheniformis* can tolerate high salt concentrations (up to 5% NaCl) and at temperatures up to 50°C (Cooper et al., 1981; Daryasafar et al., 2016). *Gordonia amicalis* is able to degrade paraffins at 40°C in the presence of 5% NaCl (Hao et al., 2008). *Marinobacter hydrocarbonoclasticus*, which was isolated at the head of an oil-producing well in Vietnam, was shown to grow on high salt concentrations (5% NaCl) and degrade *n*-hexadecane, pristane and some crude oil components (Huu et al., 1996). Ollivier et al. (1998) reported that methanogenic *Methanocalculus halotolerans* isolated from an oil well grows at the highest reported salt concentration – up to 20% NaCl and at temperatures up to 45°C.

### Facultative Anaerobic Microorganisms

As mentioned earlier, the degradation of oil in reservoirs typically occurs in the absence of oxygen (Head et al., 2010), therefore it is believed that indigenous bacteria adopt anaerobic pathways (e.g., iron reduction and methanogenesis) for hydrocarbon degradation (Head et al., 2003; Jones et al., 2008). A strain of *B. licheniformis*, isolated from Omani oil field, was able to grow under anaerobic conditions in Berea sandstone core-plugs, and was capable of degrading oil as evidenced by a measured increase in the fraction of lower-molecular-weight hydrocarbons in the oil (Al-Sayegh et al., 2015). In this study, the authors showed that after 5 days of incubation with *B. licheniformis*, the core was flooded with brine at 40°C to recover an additional 16% of original oil (Al-Sayegh et al., 2015). *G. amicalis* strain LH3 degraded paraffin by 2.3% (w/w) at a rate of 4.4 mg/d under anaerobic conditions after 10 days of cultivation (Hao et al., 2008). The strain also reduced oil viscosity by 45% and degraded oil by 10.5% (w/w) under aerobic conditions after 7 days of cultivation (Hao et al., 2008). It should be noted that oxygen can enter the reservoir through the production fluids during drilling, or in the case of shallow reservoirs, by naturally occurring faults and fractures in the rock formation that would provide migration paths for water from nearby water-saturated rock sediments (Head et al., 2014). Sampling of uncontaminated reservoirs and testing for microbial growth under a strict anaerobic environment is practically impossible. Anaerobic conditions can be simulated in the laboratory, but whether the growth of microorganisms is due to the introduction of oxygen in the reservoir or to anaerobic pathways is still very difficult to pin down due practical reasons and unavailability of controls in the field.

### Ultramicrobacteria (UMB)

When considering injection of indigenous or exogenous single bacterial species or a bacterial consortium in a reservoir to produce the desired bio-chemicals (e.g., biosurfactants), the bacteria should be smaller, or less than 20% of the size of the rock pore throats (between 0.5 and 5.0 μm) in the formation. One type of microbial group with these desirable qualities is the ultramicrobacteria (UMB), as they have a diameter of less than 0.3 μm that would permit them to penetrate into the oil-bearing formation without creating plugging problems. According to Davis and Updegraff (1954), it is imperative that the pore-entry diameter be at least twice the diameter of the microbial cells that are injected into the reservoir, otherwise plugging will occur. However, a study from 1992 implemented formation of UMB by starvation of *Pseudomonas* sp. (reduced cell size from 1.5–2.5 μm to 0.2–0.4 μm) isolated from a Canadian oil well before injecting it in a sandstone reservoir simulator along with nutrients as a method for selective plugging (Cusack et al., 1992). The simulator reservoir was incubated at 22°C to allow the bacteria to recover their cell size, and the permeability changes in the sand subsequently measured. The permeability of the sand cores changed from 3.3–5.9 d to 200 md – 5.9 d, indicating that UMB could be used for selective plugging.

In addition to their small size, UMB are also characterized by smaller genome size (from 3.2 to 0.58 Mb) and diverse morphology, physiology, biochemistry and ecology (Duda, 2011). For example, oligotrophic UMB have been isolated from marine environments (Williams et al., 2011), soil (Lysak et al., 2010), groundwater (Ross et al., 2001) among other environments. Due to their small size, UMB have some advantages such as higher metabolic activity per unit of volume of water compared to larger cells, better growth under nutrient-limited conditions facilitated by larger surface-to-volume ratio, and the ability to generate a larger biomass from a defined substrate pool (Williams et al., 2011). Some interesting UMB include the marine hydrocarbon-degrader *Cycloclasticus oligotrophus* and the thermophilic archaeon *Nanoarchaeum equitans* isolated from hydrothermal vents (Duda, 2011). Given their diverse physiology, biochemistry and ecology, it is highly possible that suitably adapted UMB also reside in petroleum reservoirs. Research and field trials using UMB for other MEOR applications other than selective plugging, however, are almost non-existent and it would seem this field of research warrants attention.

## Technological Advances in Meor

From laboratory studies conducted between the 1940s and 1980s, MEOR has progressed into a successful field application over the past two decades. The following are some of the main advances that have resulted in its successful application: (1) the stimulation of indigenous microbiota in oil reservoirs by injection of oxygen and salts together with water; (2) the injection of pre-adapted mixed enrichment cultures (AMEC); and (3) the use of UMB (formed by selective starvation) for the selective plugging of high-permeability channels. Two of the most notable recent advances in MEOR are the use of enzymes for EOR (EEOR) and the development of genetically engineered microorganisms for EOR (GEMEOR). Both of these technologies involve using microorganisms that are capable of tolerating extreme conditions within a reservoir (Nwidee et al., 2016). An example for application of genetically engineered microorganism for MEOR was demonstrated by Sun et al. (2011), who engineered a bacterial strain, called GW3-3.0, for polymer production at higher temperatures, from the biopolymer-producing organism *Enterobacter cloacae* and thermophilic strains of *Geobacillus*. The GW3-3.0 strain was used to conduct a core flood experiment to assess its suitability for MEOR with promising results.

As noted earlier, there are two main categories of *in situ* MEOR methods: aerobic bacterial systems and anaerobic bacterial systems. Achieving the desired rate of mobilization of oil in anaerobic systems will be a slow process due to the lack of oxygen in the oil-bearing formation (Sunde, 1992). If an aerobic system is to be used, then oxygen must be supplied externally. Oxygen can be usually supplied through the drilling fluids or water flooding phase of the hydrocarbon production process (Sunde, 1992). Another recent method for supplying oxygen in the reservoir was developed by the company Royal Dutch Shell, which involved the injection of perchlorate that becomes reduced by bacteria to produce oxygen *in situ* (Patent WO2011076925; Table 3). Titan Oil Recovery Inc. work on a nutrient product that alters the cell surface of microorganisms in order to induce a unique oleophilic activity, which dislodges and breaks down oil droplets to create natural emulsions (Zahner et al., 2010). The latest approach is the use of genetic engineering and recombinant DNA technologies to develop strains of bacteria with oil recovery traits and resilience to extreme environment conditions.

Table 3 provides more information on the latest technological advancements in MEOR by presenting a selection of recent patents in this specific field. Patents are particularly beneficial for small companies which may want to sell their technology to others who are in a better financial and technological position to develop the technology further. The patent history on MEOR has mainly focused on the reduction of oil viscosity, reduction of paraffin build-up in well bores, control of bio-plugging, inhibition of SRB activity and oxygen production *in situ*. In the early 2010s, patent technology for MEOR predominantly included varying combinations of indigenous microbes via injection of treated water, usually with added nutrients such as molasses, glucose and nitrogen, and oxygen (patents US2012061117, WO2013110132). Injection of chemical oxidizing agent such as hydrogen peroxide and sodium perchlorate to stimulate *in situ* oxygen production by native microorganisms, also emerged as a promising technology which was patented by Shell International Research (patent WO2011076925). However, the amount of produced oxygen *in situ* is recognized as potentially insufficient to support the desired microbial biodegradation of the heavy oil (Head et al., 2014). With the advancement of DNA sequencing technology in the early 2010s, it became possible to identify and characterize at in depth resolution the key microorganisms native to oil reservoir and understand how their metabolic pathways work. As a consequence, the MEOR technology moved on to direct injection of selected microorganisms, usually incubated in laboratory conditions, for the *in situ* production of useful metabolites like biosurfactants (patent CN104877928) and bioacids (patent US20150053407). The rationale behind this was that it would significantly reduce the costs of buying stimulating substrates, modifying existing or even installing new equipment for injection of treated water.

In the second half of the 2010s, the use of genetically engineered bacteria started to emerge as a promising technology for MEOR. One such patent (MX343588) claimed that a bioengineered halophilic microbe was capable of metabolizing only high-molecular-weight hydrocarbons without altering the short-chain alkane fraction, which was deemed a significant breakthrough considering that alkanes are generally the preferred choice of substrates by hydrocarbon-degrading microorganisms. However, the use of genetically engineered microorganisms remains highly controversial for many reasons, such as the high cost and extensive studies needed to validate their safe use and effectiveness, and as such they are not widely used in practice. The patent history on MEOR suggests that the stimulation of indigenous reservoir microorganisms remains as the most common type of technology for MEOR, and that a deeper understanding to how the reservoir communities respond to such stimulations is fundamental to advancing the success of MEOR in the future.

## Conclusion and Future Research

The process performance of MEOR remains hard to predict because influencing the environmental conditions in the reservoir to stimulate growth and/or product formation by the microorganisms is hard to achieve. As conditions vary between different reservoirs, the MEOR process must be customized for the specific conditions in each reservoir for it to be successful. At present, oil-producing companies see section “Microbial Enhanced Oil Recovery (MEOR)” as a high-risk technology to achieve efficient and predictable oil recovery. Whilst modeling approaches for predicting reliable oil recovery under simulated reservoir conditions shed a ray of promise, progress on this has been relatively quite slow. The potential development of a “universal” formulation, constituting a mixture of nutrients, selected microorganisms and (bio)surfactants in a buffering solution and each at appropriate proportions, is seen as a promising line of future research in this field (Van Hamme et al., 2003).

Appreciably, the injection of microorganisms into the reservoir via a single well will only likely affect a rather small area surrounding the wellbore, unless there are cracks in the formation. Therefore, only a very small proportion of the oil in the whole reservoir would be potentially recovered. As often reported in the literature, the apprehension of results from field studies is problematic due to the common unpredictability of the recovery process (Brown, 2010). More research is therefore needed to understand and describe the chemical, physical, mechanical, and biological variables that affect the recovery process.

Since microbial performance in laboratory experiments cannot be expected to be same as in the field, it is unrealistic to forecast the outcome of the MEOR process in the field. As mentioned previously, the microbial activity during the majority of successful single-well tests occur in the immediate area adjacent to the wellbore. This makes it difficult to determine whether the results are due to so-called well stimulation or to MEOR. Novel approaches for monitoring microbial performance *in situ* are necessary in order to determine their effectiveness in actual oil recovery. Hence, research studies that focus to understand the different factors affecting MEOR success in different reservoir settings are recommended.

Most field trials that have been performed were conducted for relatively short periods of time to conclusively demonstrate any long-term effects. Indeed, sufficient investment and expertise are required for a satisfactory field trial. In addition, the cost of developing a given MEOR treatment is high and companies would normally expect some protection of their investment, such as in the form of a patent. However, if the majority of technological advancements in MEOR are patented, this could hinder the availability of the process to everyone else in the oil industry. Technical knowledge from companies involved with development of MEOR methods, the use of field trials, development of know-how, and any other aspect of MEOR research and application should be widely accessible to all stakeholders involved in the process. In addition, the development of novel, cheaper and expedient MEOR methods are logical avenues to explore in this line of research. For every new MEOR mechanism developed, an assessment of the mass balance is essential to determine the potential oil recovery versus the amount of input materials required and whether the mass balance will be economically attractive.

Despite the fact that MEOR has been around for around 70 years, it has not been widely used by the industry, mainly because of a lack of multidisciplinary research to resolve many of the limitations or knowledge gaps that hinder its advancement. Both industry and academia recognize that in order to develop robust MEOR methods, multidisciplinary and collaborative research between both sectors is needed and that would include expertise in microbiology, petroleum engineering, petrophysics, process engineering etc. Specific research topics for further investigation should include determination of parameters relating to transport, growth and metabolite production by microorganisms in petroleum reservoirs. Several important questions remain unresolved. How much oil is lost due to adsorption to reservoir rock formation altered by injection of biosurfactants and biopolymers? What enhancement of conformance is achieved by promoting flow of water into low-permeability zones? What are the challenges in delivering and maintaining the microorganisms in the reservoir for a long period of time? What mechanisms permit certain bacteria to increase oil recovery, and how do these interact and evolve over time? What is the overall benefit of bacteria in mobilizing oil in pores of different sizes and material nature (sandstone, carbonate, limestone etc.)? What influence do biofilms have on the oil-water interface, and could the engineering of recombinant microorganisms add value to the economics of MEOR?

The development of novel metagenomics approaches in recent years has provided opportunities to characterize the indigenous microbial communities, both at a phylogenetic and functional level, in oil reservoirs to an unprecedented depth of resolution. The information that could be gained from using metagenomics approaches to understand the microbiology of oil reservoirs would be expected to uncover microbial strains that would be new to science, as well as open opportunities to harness their potential for MEOR. This coupled with biotechnological approaches (e.g., recombinant DNA techniques) could have immense potential for improving MEOR. In fact, MEOR now heavily relies on techniques based on genetic engineering, *in situ* and *ex situ* cultivation of various organisms, biosynthesis of cellular products and microbial enzyme kinetics. It is recognized that research to improve MEOR requires the discovery and isolation of new types of microbial strains (especially those that are active under anaerobic conditions and that can rapidly produce biomass or surface-active biopolymers); genetically, engineering of new bacterial strains could be made to be more efficient, such as for the *ex situ* production of useful biosurfactants and/or biopolymers, or as more efficient degraders of the high-molecular-weight hydrocarbons in heavy crude oils that would subsequently allow these oils to be more easily recoverable from reservoirs (Patel et al., 2015). However, regulatory rules and conditions upon safety do limit the release of genetically manipulated organisms into the environment. Taking into account the lack of significant advancement in MEOR technology in recent decades, and considering the global climate change challenges that presently, and for decades to come, threaten life on our planet from uncontrolled greenhouse gas emissions, future investment to developing technologies for harnessing energy from non-fossil fuel sources is a frontier that warrants significant and urgent attention.

## Author Contributions

CN sourced the information for the manuscript, and together with TG, wrote the manuscript.

## Conflict of Interest

The authors declare that the research was conducted in the absence of any commercial or financial relationships that could be construed as a potential conflict of interest.

## Footnotes

1 http://www.marisurf.eu
